# Multifocal myositis ossificans in masticatory muscles 30 years after gunshot wound: case report and literature review^☆^

**DOI:** 10.1016/j.bjorl.2016.03.018

**Published:** 2016-04-30

**Authors:** Beatriz Godoi Cavalheiro, Claúdio Roberto Cernea, Lenine Garcia Brandão

**Affiliations:** a Universidade de São Paulo (USP), Faculdade de Medicina, Instituto do Câncer do Estado de São Paulo (ICESP), São Paulo, SP, Brazil; b Universidade de São Paulo (USP), Faculdade de Medicina, Departamento de Cirurgia de Cabeça e Pescoço, São Paulo, SP, Brazil

## Introduction

Myositis ossificans is a benign, non-neoplastic condition characterized by heterotropic bone formation in muscle and other soft tissues, frequently associated with direct and acute trauma.^1^ It generally occurs as single lesions in extremities, especially inferior extremities, which are more susceptible to trauma. In the last 50 years, a few cases in the musculature of the face have been reported; the differential diagnosis includes other malignant conditions, especially when no associated trauma can be identified.

We report a case of a man who developed three lesions compatible with myositis ossificans, thirty years after suffering an injury to his face.

## Case report

A 71-year-old man was admitted with a history of trismus, pain in the upper left labio-gingival sulcus and difficulty in adjusting his partial denture for the past 20 days. He related a gunshot wound suffered around 30 years ago, with an entry point in his right cheek and no exit wound. At the time, he was treated by a trauma service and submitted to facial fracture fixations and bone grafts in the palate and alveolar ridge. History also included non-insulin dependent diabetes mellitus, high blood pressure and a prostatectomy eight years prior for prostate cancer. Laboratory analysis demonstrated normal fidings on serum calcium, alkaline phosphatase, parathyroid hormone levels and renal function.

He was in excellent overall health, with a cheek bulge on the left and discrete associated cutaneous hyperemia. Trismus was observed, limiting mouth opening 2 cm. A bulge in the cheek mucosa was also observed near the labiogingival sulcus, with intact oral mucosa, where a 1.5 cm and well delimited firm lesion, fixed to deep structures, could be felt.

**Figure 1:**
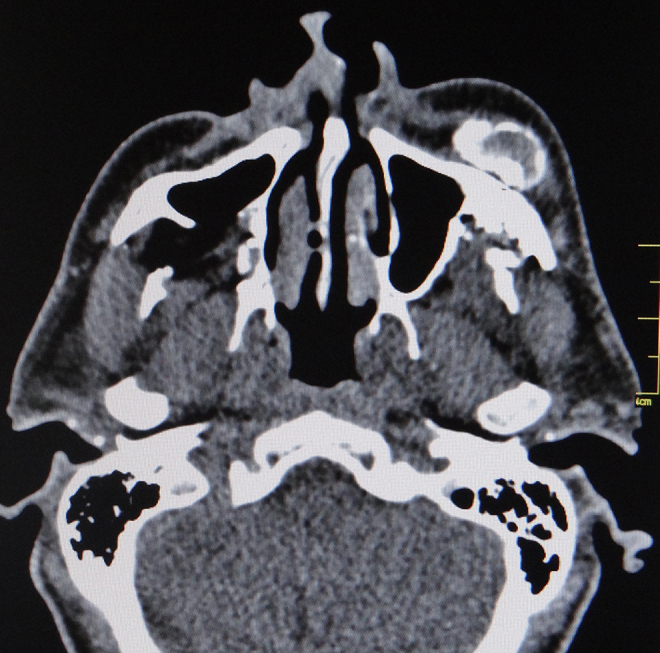
CT scan. Identification of the largest lesion, with a radiolucent interior and compact bone periphery and bulging of adjacent soft tissues.

**Figure 2:**
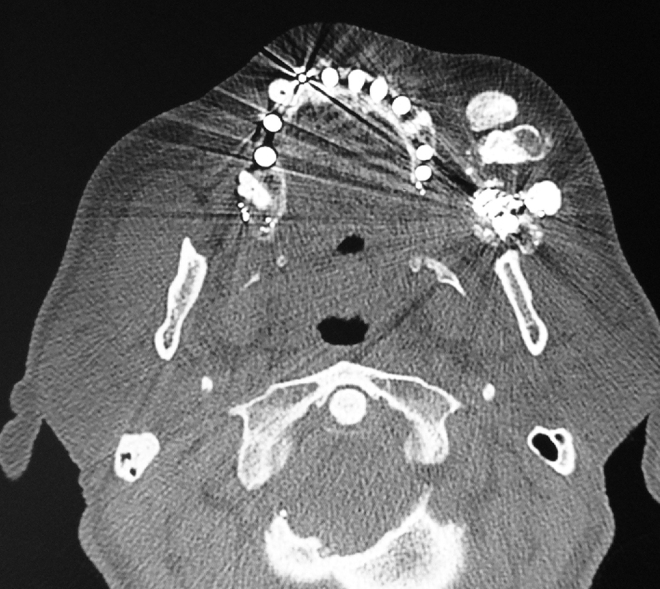
CT scan, bone window. View of three bone lesions and metallic fragments.

When submitted to computerized tomography scan of the face, a metallic fragment was identified next to the left masticator and buccal spaces, lateral to the maxilla, in addition to metallic fragments adjacent to the zygomatic arch and in the masticator space. Deformities were identified in the walls of the ipsilateral maxillary sinus with bone loss and healed fractures. Metallic fragments were also found in the right infratemporal fossa with a fracture of the posterior wall of the maxillary sinus, bone loss in the palate and anterior wall of the left maxillary sinus and sclerosis of the zygomatic arch on the same side. Three dense amorphous ossified formations were observed in the subcutaneous tissue of the maxillary region and left masticator space, amongst the temporal, masseter and mimetic musculature, in a cortical/medullary pattern, the largest measuring 3.5 cm × 2.5 cm × 1.5 cm (Figure 1, Figure 2). Lymph nodes were observed at cervical levels I and II on the right.

**Figure 3:**
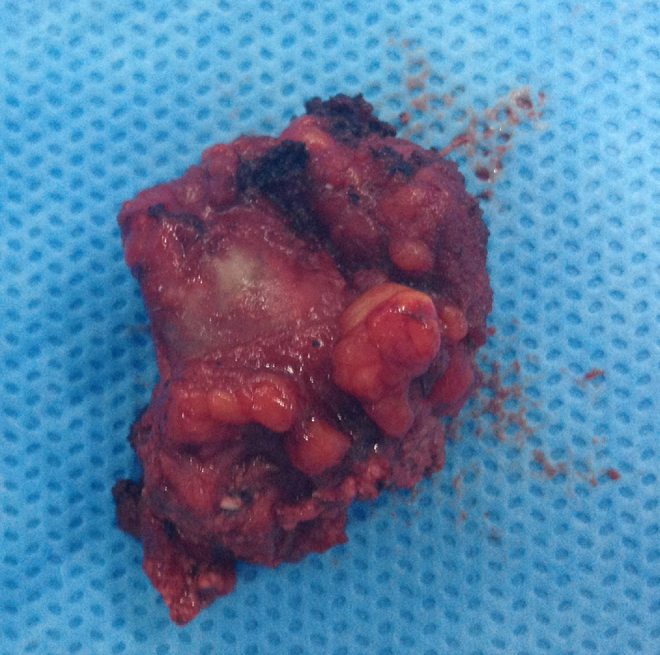
Whitish bone lesion associated with the adjacent inflammatory process.

The lesions were removed through a mucosal incision on the left superior labiogingival mucosa, revealing structures that were hardened and firmly attached to adjacent tissues (Fig. 3). Projectile fragments were also removed from the pterygoid musculature, adjacent to the temporomandibular joint.

Immediately after surgery the patient experienced a reduction in trismus, despite a progression of paresis of ophthalmic and buccal branches of the facial nerve and the mimetic musculature, expected due to extensive surgical manipulation.

A pathological examination identified fragments of compact bone tissue amidst the fibrin and hemorrhaging with fibroplasia and mixed inflammatory infiltrate in periosteal tissues, with no signs of malignancy. Findings were compatible with myositis ossificans.

Two months after the operation, an infection occurred in the soft tissues of the left hemiface, probably due to contamination via the maxillary sinus, which improved after antibiotic therapy. The patient has been receiving speech therapy for one year, since the operation, with a substantial reduction in trismus and partial recovery of neural function.

## Discussion

We describe a case of a circumscript myositis ossificans or traumatic myositis ossificans, unlike the progressive type – a rare genetic condition that is incurable. The former may be associated or, less frequently, unassociated with trauma. The associated trauma can be perforating or closed, burns, infections, fractures, neurological traumas, etc.^2^ It is uncommon in the face and reports described the involvement of masticatory muscles, such as the masseter, buccal, pterygoid and temporal muscles. Concerning its etiology in these muscles, the most common causes described are tooth extraction, local anesthetic injection, migrating odontogenic abscess, cervical collar, genioplasty, complicated orthodontic treatment, direct force and facial skeleton fractures.^3^

Symptoms include an increase in volume and temperature, erythema, pain, paresthesia and restricted local mobility when the affected area is associated with a joint.^2^ When it occurs in the area of the face, the masticatory muscles are most often affected, as observed in the case reports, and the most prevalent clinical fiding is a progressive limitation of motion in the mandible (trismus).^4^, ^5^

Jiang et al.^4^ hypothesized that infection and trauma exhibit an equally important role in the pathogenesis of myositis ossificans in the masticatory muscles. In the present case, the patient came to us with an acute facial cellulitis that could be related to the discontinuity of the maxillary sinus anterior wall and consequent local infection. However, for the past 30 years he never complained of such symptom or reported sinus events. In turn, the inflammatory signs developed after the trismus and difficulty in adjusting the partial denture, so the bone formation would not be expected to be associated with this acute infection. As mentioned, inflammatory signals are also part of the clinical picture, especially if there is involvement of a joint. Another point to be considered is the reconstructive operation carried out at the time of the gunshot. It also could be associated to the pathogenesis of the myositis ossificans, although the intensity of the initial trauma seems to be more pertinent to the development of the related condition.

Most cases of circumscript myositis ossificans are self-limited, in which lesions tend to regress spontaneously.^6^ It is believed that their developments are triggered by tissue necrosis or hemorrhage followed by an intense vascular and fibroblastic repair process with subsequent ossification.^7^ Initially the lesion, richly vascularized, is predominantly composed of fibroblasts with high mitotic activity. Over a period of three to six weeks the bone formation becomes evident at the edge of the lesion, like cortical bone, organized with a cortex and medullary space. The lesion generally matures after five or six months, when it may begin to regress.^6^

Computerized tomography is the imaging exam of choice for the diagnosis of this entity. Magnetic resonance is a more sensitive way of identifying small and initial lesions, though less specific.^7^, ^8^ In a mature lesion, tomography shows a zone of peripheral bone maturation with a radiolucent center.^7^ Another radiolucent zone generally separates the bone lesion from the adjacent tissues, which may aid in the differential diagnosis with invasive malignant neoplasms.^6^

Its initial differential diagnosis includes inflammatory and/or infectious processes, such as thrombosis, cellulitis and osteomyelitis.^2^ Neoplasms are also considered in the differential diagnosis, including synovial sarcoma, soft tissue sarcoma, osteochondroma, osteosarcoma, rhabdomyosarcoma^8^ and metastatic disease.^7^

Once we established an indication for surgical resection of the lesion due to symptoms and limitations in buccal opening, we did not perform biopsies. We relied on signs of myositis ossificans from the imaging. However, some authors^8^ recommend biopsies, especially when relying on clinical observation or in the absence of a precise clinical or imaging diagnosis. It is important to remember that the aspiration or biopsy of the central portion of the lesion, normally very cellular, may confuse the diagnosis. The biopsy should instead focus on the periphery of the structure,^6^ although its bone component may hamper fine-needle aspiration, the method of choice for biopsies in the face and neck region. Neither biopsy by aspiration nor frozen section examination generally provide a definitive diagnosis.^6^

Surgical resection^7^, ^9^, ^10^ and conservative treatment^2^, ^8^ are indicated. The latter is based on the possibility of spontaneous regression of the lesion, where clinical observation, rest, ice and physical therapy are recommended. Some recommend the administration of anti-inflammatory drugs and even low doses of external radiation therapy,^2^ but we do not promote this course of action. It is believed that surgical resection should be carried out after the lesion matures to avoid possible recurrence.^2^, ^9^ Early surgical excision is indicated, in turn, when the associated lesion is associated with a joint, which would lead to ankylosis by limiting movement.^2^ Diagnostic uncertainty in the presence of symptomatic or fast growing lesions is suitable for resection at the time of presentation. The majority of the consulted reports of this clinical condition in face had its patients operated on, as we did, because of the related symptons, mainly pain and trismus. Recurrence post complete surgical treatment is not a frequent condition, although the reports are limited in relation to follow up information.

Two aspects required our attention in this case. First, the development of the lesions 30 years after an injury is uncommon, since myositis ossificans is characterized by its development weeks after the trauma. We found one report of myositis ossificans in temporal muscle, 25 years after a severe trauma^9^ and another case report that related a blow to the left side of the face of a young woman “several years” before the symptoms.^5^ Mashiko et al.^10^ also reported the case of a man who suffered repeated physical abuses in the past 15 years and that could be related to the development of myositis ossificans in the masseter muscles. The second aspect was the presence of three lesions, instead of a single lesion as reported in almost all of the cases described in the specialized literature. In the last 15 years, in turn, three reports described cases of bilateral lesions.^8^

## Conclusion

In the last three years it could be observed an interesting increase in the reports in the literature of myositis ossificans in head and neck territory. Even so, this is an infrequent situation and physicians must remain alert to it, as well as the possible differential diagnoses, such as sarcomas, to ensure proper treatment and follow up. There are few reports of similar cases in muscles of the face that develop years after the trauma. There are also few reports of multiple lesions. Attention must also be made to the clinical history of the patient, looking for any possible local trauma.

## Conflicts of interest

The authors declare no conflicts of interest.
